# Leptin promotes VEGF-C production and induces lymphangiogenesis by suppressing miR-27b in human chondrosarcoma cells

**DOI:** 10.1038/srep28647

**Published:** 2016-06-27

**Authors:** Wei-Hung Yang, An-Chen Chang, Shih-Wei Wang, Shoou-Jyi Wang, Yung-Sen Chang, Tzu-Ming Chang, Shao-Keh Hsu, Yi-Chin Fong, Chih-Hsin Tang

**Affiliations:** 1 Department of Orthopedic Surgery, Taichung Hospital, Ministry of Health and Welfare, Taichung, Taiwan; 2 School of Chinese Medicine, China Medical University, Taichung, Taiwan; 3 Department of Nursing, National Taichung University of Science and Technology, Taichung, Taiwan; 4 Graduate Institute of Biotechnology, National Chung Hsing University, Taichung, Taiwan; 5 Institute of Biomedical Sciences, National Chung Hsing University, Taichung, Taiwan; 6 Department of Medicine, Mackay Medical College, New Taipei City, Taiwan; 7 Department of Orthopedic Surgery, Chang-Hua Hospital, Ministry of Health and Welfare, Puhsin Township, Changhua County, Taiwan; 8 Department of Orthopedic Surgery, Tungs’ Taichung Metroharbor Hospital, Taichung, Taiwan; 9 Department of Sports Medicine, College of Health Care, China Medical University, Taichung, Taiwan; 10 Department of Orthopedic Surgery, China Medical University Beigang Hospital, Yunlin County, Taiwan; 11 Graduate Institute of Basic Medical Science, China Medical University, Taichung, Taiwan; 12 Department of Pharmacology, School of Medicine, China Medical University, Taichung, Taiwan; 13 Department of Biotechnology, College of Health Science, Asia University, Taichung, Taiwan

## Abstract

Chondrosarcoma is the second most frequently occurring type of bone malignancy that is characterized by the distant metastasis propensity. Vascular endothelial growth factor-C (VEGF-C) is the chief lymphangiogenic mediator, and makes crucial contributions to tumor lymphangiogenesis. Leptin is an adipocytokine and has been indicated to facilitate tumorigenesis, angiogenesis and metastasis. However, the effect of leptin on VEGF-C regulation and lymphangiogenesis in human chondrosarcoma has hugely remained a mystery. Our results showed a clinical correlation between leptin and VEGF-C as well as tumor stage in human chondrosarcoma tissues. We further demonstrated that leptin promoted VEGF-C production and secretion in human chondrosarcoma cells. The conditioned medium from leptin-treated chondrosarcoma cells induced lymphangiogenesis of human lymphatic endothelial cells. We also found that leptin-induced VEGF-C is mediated by the FAK, PI3K and Akt signaling pathway. Furthermore, the expression of microRNA-27b was negatively regulated by leptin via the FAK, PI3K and Akt cascade. Our study is the first to describe the mechanism of leptin-promoted lymphangiogenesis by upregulating VEGF-C expression in chondrosarcomas. Thus, leptin could serve as a therapeutic target in chondrosarcoma metastasis and lymphangiogenesis.

Human chondrosarcoma is the second most frequently occurring type of bone malignancy which chiefly occurs in adults over 40 years of age^1^. Chondrosarcoma has been identified as the invasive and pathologically diverse malignant tumor with poor disease progression^2^,^3^. Currently, the surgical resection is a major treatment of chondrosarcoma, due to conventional radiotherapy and chemotherapy are mostly invalid. The relapse usually occurs following surgical resection since the potential for metastatic propensity. The need for a specific targeted therapy to impede the metastasis of chondrosarcoma remains urgent^4^.

Metastasis is the primary cause of cancer death worldwide. The initial stage of metastasis in most human cancer is metastatic spread to sentinel lymph nodes^5^,^6^. Tumors can promote the production of lymphatic vessels via secretion of lymphangiogenic factors, and that tumor lymphangiogenesis has been implicated in the correlation with lymph node metastasis in many types of human cancer^7^,^8^. Vascular endothelial growth factor-C (VEGF-C) is most important lymphangiogenic mediator, acting predominantly through VEGF receptor-3 (VEGFR-3) that is specifically expressed in lymphatic endothelial cells (LECs). The VEGF-C and VERFR-3 interaction has been reported to mediate LECs proliferation, survival, migration and tube formation during lymphangiogenic process^9^. Recent studies have revealed that tumor cells secreted VEGF-C plays a key role during lymphatic metastasis and tumor-associated lymphangiogenesis^6^. Moreover, clinical evidences suggest the existence of a relationship between tumor expressing VEGF-C and the disease progression of cancer in various tumor types, including melanoma, pancreatic, breast, colorectal and lung cancer^10^,^11^,^12^,^13^,^14^. Blockade of tumor-mediated lymphangiogenesis has been reported to markedly inhibit cancer metastasis. Therefore, the identification of mechanisms underlying VEGF-C-mediated lymphangiogenesis is necessary for discovering novel prognostic and therapeutic strategies of cancer^15^,^16^.

MicroRNAs (miRNAs) are small noncoding RNAs molecules that interfering with the translation or stability of target transcripts^17^,^18^. They integrating to the 3′untranslated region (3′UTR) of mRNA and regulate gene expression through complementary base pairing^19^,^20^. Increasing studies have reported that miRNAs control progression and metastasis of human cancer cells. miRNAs have been proposed to intervene numerous functions of cancer cells, including survival, apoptosis, autophagy, migration, invasion, angiogenesis and lymphangiogenesis^21^. Several investigations demonstrate that miRNAs inhibit lymphangiogenesis and tumor dissemination through the dysregulation of miR/VEGF-C signaling^22^,^23^. miR-128 has been reported to inhibit lymphangiogenesis in human lung cancer cells by directly suppressing VEGF-C expression^24^. miR-206 also abrogates the expression and secretion of VEGF-C, and subsequently inhibits tumor lymphangiogenesis in pancreatic cancer^25^. Furthermore, miR-101 has been documented to suppress migration and invasion via negatively regulating VEGF-C expression in bladder cancer and cholangiocarcinoma cells, respectively^26^. Nevertheless the effect of miRNA in regulating VEGF-C production in human chondrosarcoma cells is poorly understood.

Leptin, 16 kDa product of *ob* gene, is secreted and expressed by adipocytes which is interacted with leptin receptor (OBR)^27^. Compelling evidences indicate that leptin is associated with tumourigenesis and metastasis in several types of cancer^28^,^29^. We previously reported that leptin enhances cell migration through activation of integrin αvβ3 and increases VEGF-A-dependent tumor angiogenesis in human chondrosarcoma^30^,^31^, implying that leptin is involved in the metastasis of chondrosarcoma. However, it is still not well-recognized whether leptin increases VEGF-C expression to facilitate tumor-associated lymphangiogenesis in human chondrosarcoma. In present study, we examined the effect of leptin in VEGF-C-mediated lymphangiogenesis, and evaluated the involvement of miRNA in human chondrosarcoma cells.

## RESULTS

### Leptin and VEGF-C display a significant crosstalk in human chondrosarcoma tissues

**Figure 1:**
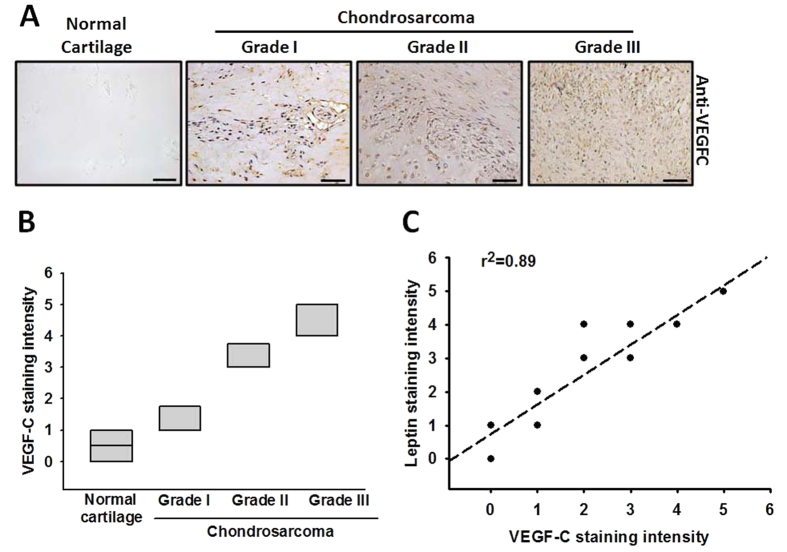
The leptin and VEGF-C expression in normal cartilage and chondrosarcoma patients. (**A**) Human normal cartilages or chondrosarcoma tissues were IHC stained with anti-VEGF-C antibody. The quantitative data are shown in (**B**,**C**) The correlation of leptin, VEGF-C and tumor stage.

Our previous reported that leptin facilitates tumor metastasis and angiogenesis in human chondrosarcoma^30^,^31^. We indicated that the leptin expression is highly correlated with tumor stage according to the IHC analysis of human chondrosarcoma tissues. To characterize the role of leptin in tumor lymphangiogenesis of chondrosarcoma, we first analyzed the expression profile of VEGF-C in specimens of chondrosarcoma patients. The VEGF-C expression was higher in tumor specimens than in normal tissues (Fig. 1A). Accordingly, the high level of VEGF-C expression correlated significantly with tumor stage (Fig. 1B). We quantitated the IHC results and found the leptin and VEGF-C expression have high positive relationship in human chondrosarcoma patients (Fig. 1C). These results suggest that leptin is strongly associated with VEGF-C expression and tumor stage in chondrosarcoma patients.

### Leptin induces VEGF-C-mediated lymphangiogenesis

**Figure 2:**
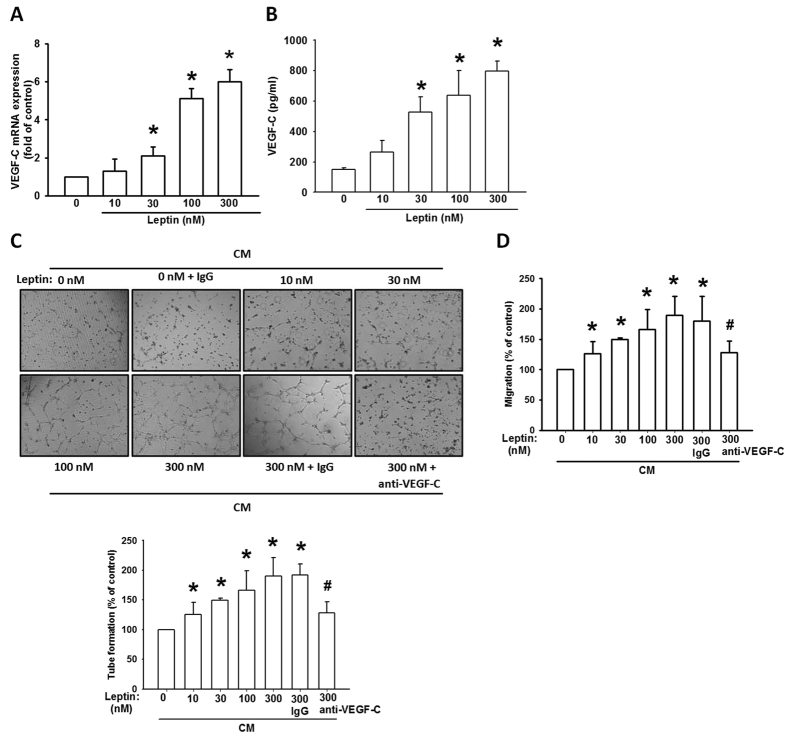
Leptin promotes lymphangiogenesis by VEGF-C production in human chondrosarcoma. JJ012 cells were stimulated with leptin for 24 h, the VEGF-C mRNA level and protein expression in culture medium were measured by qPCR (**A**) and ELISA (**B**). JJ012 cells were stimulated with leptin for 24 h, or preincubated with IgG control antibody or VEGF-C antibody (1 μg/mL) for 30 min then incubated with leptin (300 nM) for 24 h. The incubated media were collected and defined as conditioned medium (CM). The CM were added to LECs and examined tube formation activity (**C**). The CM were also added into the lower chamber of Transwell. The LECs were applied in the upper chamber and the migrated LECs was quantified (**D**). The quantitative results were expressed as mean ± SEM. *P < 0.05 as compared with control group; ^#^*P* < 0.05 as compared with leptin-treated group.

Next we examine the effects of leptin in VEGF-C production and lymphangiogenic process. Incubation of a chondrosarcoma cell line (JJ012 cells) increased VEGF-C mRNA expression and protein secretion (Fig. 2A,B). In addition, leptin also promotes other VEGF families including VEGF-A (our previous report has been documented)^30^ and VEGF-B expression (Supplementary Fig. S1). To observe whether leptin-dependent VEGF-C expression promoted lymphangiogenesis, the migration and tube formation activity in LECs were examined^32^. The conditioned medium (CM) from leptin-stimulated JJ012 cells increased migration and tube formation activity in LECs (Fig. 2C,D). Conversely, VEGF-C mAb but not IgG control abolished leptin-mediated effects (Fig. 2C,D), implying that leptin promotes lymphangiogenesis through a VEGF-C-dependent pathway.

### Leptin promotes VEGF-C expression via the FAK/PI3K/Akt pathway

**Figure 3:**
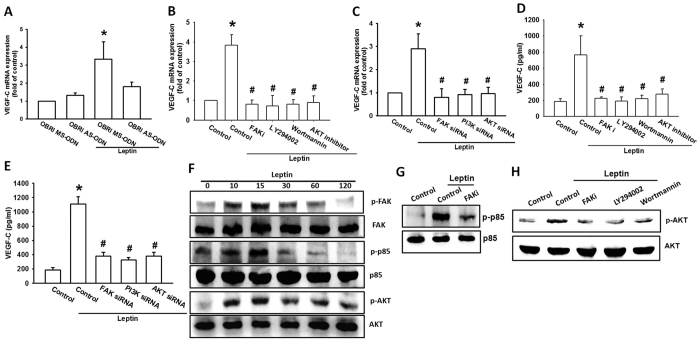
The FAK/PI3K/Akt pathway is mediated by leptin-induced VEGF-C expression. (**A**) JJ012 cells were transfected with oligonucleotide against OBRl for 24 h then incubated with leptin for 24 h. The VEGF-C mRNA level was measured by qPCR. JJ012 cells were pretreated with indicated pharmacological inhibitors or pretransfected with indicated siRNAs then incubated with leptin for 24 h. The VEGF-C mRNA level and protein expression in culture medium were measured by qPCR (**B**,**C**) and ELISA (**D**,**E**). JJ012 cells treated with leptin for the indicated time were applied to western blotting with antibodies against FAK, PI3K, and Akt pathway (**F**). JJ012 cells pretreated with indicated pharmacological inhibitors then incubated with leptin for 15 min were applied to western blotting with antibodies against PI3K (**G**) and Akt (**H**) pathway. The quantitative results were expressed as mean ± SEM. *P < 0.05 as compared with control group; ^#^*P* < 0.05 as compared with leptin-treated group.

Long form OBR receptor (OBRl) has been reported to mediate leptin-induced chondrosarcoma metastasis and angiogenesis^30^,^31^. Transfection of JJ012 cells with OBRl AS-ODN but not MM-ODN inhibited leptin-increased VEGF-C expression (Fig. 3A), indicating OBRl involved leptin-increased VEGF-C production in human chondrosarcoma cells. Focal adhesion kinase (FAK) is recently been implicated in tumor progression processes such as angiogenesis, lymphangiogenesis and metastasis^33^. Pretreatment with FAK inhibitor or FAK siRNA transfection reversed the leptin-enhanced the expression of VEGF-C (Fig. 3B–E). Besides, leptin also increased the phosphorylation of FAK time-dependently (Fig. 3F).

PI3K/Akt is a downstream pathway in FAK signaling^34^. We therefore studied whether leptin also activates PI3K/Akt signaling pathway. Similarly, PI3K inhibitors (LY294002 and wortmannin) and p85 siRNA or Akt inhibitor and Akt1 siRNA abolished leptin-increased VEGF-C expression (Fig. 3B–E). PI3K and Akt phosphorylation were increased after leptin treatment (Fig. 3F). Conversely, pretreatment with FAK inhibitor markedly diminished leptin-induced p85 phosphorylation (Fig. 3G). Furthermore, FAK inhibitor, LY294002 and wortmannin also reduced leptin-promoted phosphorylation of Akt (Fig. 3H). These results indicated that leptin enhances VEGF-C production in chondrosarcoma via the FAK, PI3K and Akt pathways.

### Leptin enhances VEGF-C production and lymphangiogenesis by down-regulating miR-27b

**Figure 4:**
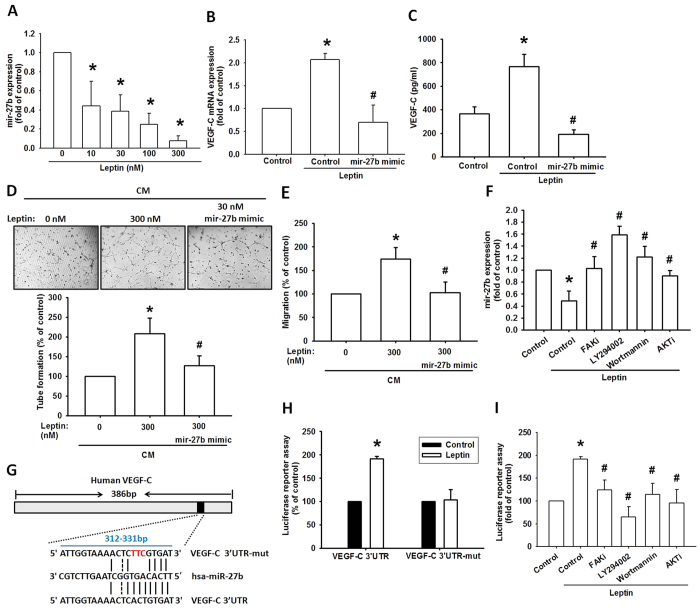
Leptin promotes VEGF-C via downregulation of miR-27b. (**A**) JJ012 cells were treated with leptin for 24 h and the miR-27b was detected by qPCR. JJ012 cells were pretransfected with indicated miRNA then incubated with leptin for 24 h. The VEGF-C mRNA level and protein expression in culture medium were measured by qPCR (**B**) and ELISA (**C**). The CM were added to LECs and examined tube formation activity (**D**). The CM were also added to Transwell and examined LECs migration activity (**E**). JJ012 cells pretreated with indicated pharmacological inhibitors then incubated with leptin were applied to qPCR for miR-27b expression (**F**). Schematic the target site of miR-27b on VEGF-C 3′UTR (**G**). JJ012 cells transfected with VEGF-C promoter-luciferase plasmids and then treated with leptin (**H**) or pretreated with indicated pharmacological inhibitors next incubated with leptin for 24 h (**I**) were subjected to luciferase activity assay. The quantitative results were expressed as mean ± SEM. *P < 0.05 as compared with control group; ^#^*P* < 0.05 as compared with leptin-treated group.

Emerging studies have indicated that miRNAs are important regulators of lymphangiogenesis and VEGF-C expression during cancer progression^21^,^22^. miRNA target prediction using open-source software (www.TargetScan.org and www.microrna.org) revealed that the 3′UTR region of VEGF-C mRNA harbors potential binding sites for miR-27b. Exogenous leptin reduced the expression of miR-27b concentration-dependently (Fig. 4A). To explore miR-27b involvement in leptin-induced VEGF-C and lymphangiogenesis, miR-27b mimic was used; transfection with miR-27b mimic diminished leptin-induced VEGF-C expression (Fig. 4B,C). On the other hand, transfection with miR-27b mimic enhanced miR-27b expression (Supplementary Fig. S2). Conversely, miR-27b mimic also diminished leptin-promoted LECs migration and tube formation (Fig. 4D,E). Furthermore, FAK inhibitor, LY294002, wortmannin and Akt inhibitor reversed leptin-inhibited miR-27b expression (Fig. 4F), indicating that leptin increases VEGF-C production and lymphangiogenesis by suppressing miR-27b expression via the FAK, PI3K and Akt pathways.

Next we study whether miR-27b manages the 3′UTR region of VEGF-C, the wild-type and mutant binding site of VEGFC-3′UTR luciferase plasmids were used (Fig. 4G). The results show that leptin increased luciferase activity in the wt-VEGFC-3′UTR plasmid (Fig. 4H). Nevertheless, leptin did not affect the luciferase activity in the mt-VEGFC-3′UTR plasmid (Fig. 4H). In addition, treatment with FAK inhibitor, LY294002, wortmannin and Akt inhibitor diminished leptin-promoted wt-VEGFC-3′UTR luciferase activity (Fig. 4I), suggesting that miR-27b inhibites the protein expression of VEGF-C via integrating to the 3′UTR region of the human *VEGF-C* gene through FAK, PI3K and Akt pathways.

### Inhibiting leptin expression suppresses lymphangiogenesis *in vivo*

**Figure 5:**
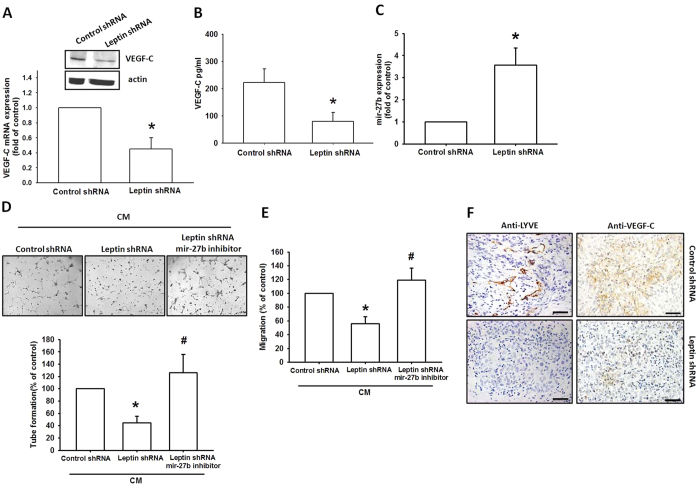
Leptin knockdown decreases lymphangiogenesis *in vivo.* The expression of VEGF-C and miR-27b in indicated cells were analyzed by qPCR (**A**,**C**), western blotting (**A**) and ELISA (**B**). The CM were added to LECs and examined tube formation activity (**D**). The CM were also added to Transwell and examined LECs migration activity (**E**). After 28 days injected with indicated cells in mice, the tumors were embedded in paraffin and sections were immunostained using LYEC and VEGF-C antibodies. The quantitative results were expressed as mean ± SEM. *P < 0.05 as compared with control group; ^#^*P* < 0.05 as compared with leptin-treated group.

Here, we found that leptin promoted VEGF-C expression in chondrosarcomas and enhanced LECs lymphangiogenesis. It is critical to pinpoint the role of leptin *in vivo*. Previously, we established JJ012 cells stably expressing leptin shRNA, in which we found that the expression of leptin was decreased in leptin shRNA stable clones^30^. In this study, leptin knockdown significantly reduced the expression of VEGF-C (Fig. 5A,B) and increased miR-27b expression (Fig. 5C). CM collected from JJ012/control shRNA promoted LEC cell migration and tube formation, but this activity was decreased during incubation with CM collected from JJ012/leptin shRNA (Fig. 5D,E). In addition, transfection with miR-27b inhibitor rescued leptin shRNA-inhibited LEC cell migration and tube formation (Fig. 5D,E). We also previously found that leptin knockdown reduced tumor growth in mice compared with the JJ012/control shRNA group^34^. Here, we used IHC staining to examine the level of lymphangiogenesis. Analysis revealed that leptin knockdown impedes the expression of lymphatic markers LYEC and VEGF-C (Fig. 5F) and inhibits lymphangiogenesis *in vivo*.

## DISCUSSION

**Figure 6:**
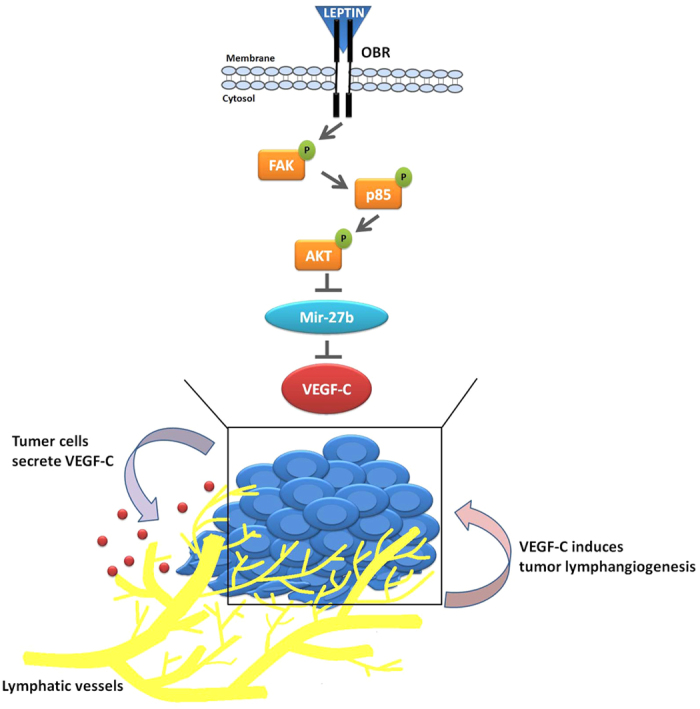
Schema of leptin promotes lymphangiogenesis in chondrosarcomas. Leptin promotes VEGF-C production by inhibiting miR-27b expression via the OBRl, FAK, PI3K and Akt pathways. Leptin-induced VEGF-C production subsequently recruiting LECs to the chondrosarcoma microenvironment and promoting lymphangiogenesis.

Although chondrosarcoma is a relatively rare human cancer, the notorious aggressiveness of chondrosarcoma is due to its metastatic potential and poor prognosis^4^. Lymphangiogenesis is an indispensable step for cancer metastasis, facilitating cancer development by the generation of new lymphatic vessels^5^. Accumulating evidences demonstrate that increased levels of VEGF-C promotes tumor relapse and poor prognosis, and thus VEGF-C represents a potential target for preventing lymphatic metastasis^6^,^15^. Here we indicate the clinical significance of leptin and VEGF-C in specimens of chondrosarcoma patients. In summary, we show that leptin increases the expression and secretion of lymphangiogenic factor VEGF-C by down-regulating miR-27b via FAK, PI3K, and Akt pathways in human chondrosarcoma cells (Fig. 6), and thereby promotes lymphangiogenesis in human LECs, indicating that leptin and miR-27b may be the novel molecular targets to restrict VEGF-C-mediated lymphangiogenesis in chondrosarcoma microenvironment.

The first step of metastasis is cancer cells invasion to lymphatic system^7^. The lymphatic endothelium, which comprises LECs, is a specialized endothelium and is distinct from the vascular endothelium^35^. Tumor lymphatic vessels serve as a pivotal route for metastatic cancer cells, due to their leaky nature and secretion of tumor-recruiting factors^9^. More understanding of molecular mechanisms underlying tumor lymphangiogenesis will provide new insights in the process of metastasis. However, the study of lymphangiogenesis has been impeded by the difficulties in the isolation and propagation of LECs from different organs^36^,^37^. To conquer the above limitations, we used a “conditionally immortalized” of human LECs cell line, which transformed with the human telomerase reverse transcriptase (hTERT), and maintain their ‘lymphatic’ endothelial characteristics after repeated passages. This immortalized human LECs keep the ability to sprout, elongate, migrate and reorganize to form the capillary-like tube structure within 4–8 h, a process called tube formation, and this function of LECs represents the major process of lymphangiogenesis. In this study, we found that CM from leptin-treated cells profoundly stimulated tube formation of human LECs. On the contrary, knockdown of leptin suppressed CM-induced LECs tube formation. These results provide evidences that leptin-mediated VEGF-C production induces lymphangiogenesis *in vitro*. Furthermore, we found that levels of leptin and VEGF-C in clinical specimens from patients with chondrosarcoma were correlated with tumor stage, implying that leptin may be a candidate prognostic indicator for chondrosarcoma progression. These findings support the notion that leptin and VEGF-C might serve as promising targets for therapeutic intervention to block cancer progression and metastasis.

Evidence indicates that FAK, a potential candidate signaling molecule, mediates cancer metastasis^38^. Here, we report that both FAK inhibitor and siRNA antagonized leptin-promoted the production of VEGF-C. Incubation of chondrosarcoma cells with leptin increased phosphorylation of FAK, suggesting that FAK activation plays a crucial role in leptin-increased VEGF-C production and lymphangiogenesis. Conversely, PI3K/Akt activation is an important downstream event of FAK signaling^39^. In the current study, inhibition of PI3K and Akt by pharmacologic inhibitors or genetic siRNAs reduced VEGF-C production. We also found that leptin enhanced PI3K and Akt phosphorylation, and was inhibited by FAK inhibitor. These findings show that FAK-dependent PI3K/Akt pathway play a key role in leptin-increased VEGF-C expression and lymphangiogenesis.

Small non-coding miRNAs, the average length of approximately 18 to 22 nucleotides, negatively regulate gene expression by either translational repression or mRNA cleavage through integrating to 3′UTR sequences of goal mRNA^17^,^21^. Inhibited biogenesis of miRNAs has been widely observed in human cancer^22^. Accumulating evidences further indicate that numerous miRNAs can impede cancer progression via direct suppression of VEGF-C. miR-101, miR-128, miR-206 and miR-1826 have been documented to reduce tumor growth, lymphangiogenesis and metastasis by targeting VEGF-C in a variety of human cancer cells^24^,^25^,^26^,^40^,^41^,^42^. Current study showed that leptin markedly repressed miR-27b expression in human chondrosarcoma cells *in vitro* and *in vivo*. Transfection with miR-27b mimic antagonized leptin-induced VEGF-C production and LECs tube formation. Strikingly, we revealed that miR-27b directly inhibited protein production of VEGF-C through binding to the 3′UTR of the human *VEGF-C* gene, thereby negatively regulating VEGF-C-upregulated lymphangiogenesis. Thus, these findings provide information on the potential miRNA-based molecular diagnosis and treatment for VEGF-C-mediated tumor lymphangiogenesis.

## Materials and Methods

### Materials

Rabbit monoclonal antibodies specific for p85, p-p85, Akt, p-Akt, FAK, and FAK as well as anti-mouse and anti-rabbit IgG-conjugated horseradish peroxidase were purchased from Santa Cruz Biotechnology (Santa Cruz, CA, USA). Rabbit monoclonal antibodies specific for VEGF-C and control IgG were purchased from Abcam (Cambridge, MA, USA). The ELISA kit for VEGF-C was obtained from PerpoTech (Rocky Hill, NJ, USA). The recombinant human VEGF-C was obtained from R&D Systems (Minneapolis, MN, USA). The human chondrosarcoma tissue array was obtained from Biomax (Rockville, MD, USA). The Matrigel was purchased from BD Biosciences (Bedford, MA, USA). The OBRl antisense and missense oligonucleotide (ODN) were purchased from MDBio (Taipei, Taiwan)^31^. The Trizol, Lipofectamine 2000, MMLV RT kit, miR-27b mimic, miR-27b inhibitor and control miRNA were obtained from Invitrogen (Carlsbad, CA, USA). The siRNAs against p85, Akt1, FAK, and control were obtained from Dharmacon Research (Lafayette, CO, USA). The TaqMan assay kit and TaqMan MicroRNA Reverse Transcription Kit were obtained from Thermo Fisher Scientific (Grand Island, NY, USA). LY294002 and other pharmacological inhibitors were purchased from Sigma-Aldrich (St. Louis, MO, USA)

### Cell culture

The human chondrosarcoma cells line (JJ012) was obtained from Dr. Sean P. Scully (University of Miami School of Medicine, Miami, FL). Cells were maintained in humidified air containing 5% CO_2_ at 37 °C with Dulbecco’s modified Eagle’s medium (DMEM)/α-minimum essential medium (MEM), 10% fetal bovine serum (FBS), 100 units/ml penicillin and 100 μg/ml streptomycin (Gibco-BRL Life technologies; Grand Island, NY, USA)

The human telomerase-immortalized human dermal lymphatic endothelial cells (hTERT-HDLECs), an immortalized human LEC line, was purchased from Lonza (Walkersville, MD, USA). These immortalized human LECs represent CD31 positive/podoplanin positive, and retain their ability to uptake acetylated LDL and induce tube formation. The human LECs were grown in EGM-2 MV BulletKit Medium consisting of EBM-2 basal medium plus SingleQuots kit (Lonza). Cells were seeded onto 1% gelatin-coated plastic ware and cultured at 37 °C with 5% CO_2_. We obtained the cryopreserved human LECs line from Lonza as passage 1, and maintained these cells according to manufacturer’s instructions as well as used between passages 5 and 10 for experiments described herein.

### Collection of conditioned medium and ELISA assay

JJ0112 cells were stimulated with leptin or pretreated with pharmacological inhibitors for 30 min or pretransfected with siRNA or miR-27b mimic for 24 h. Cells were then incubated with serum-free medium for 2 days. The medium was collected as conditioned medium (CM) and examined the expression of VEGF-C by VEGF-C ELISA kit according to the procedure described by the manufacturer.

### LECs tube formation assay

LECs were resuspended at a density of 5 × 10^4^/100 μL in culture medium (50% EGM-2 MV BulletKit Medium and 50% chondrosarcoma cell CM) and added to the 48-well plates which pre-coating with 150 μL Matrigel. LECs tube formation was photographed after 6 h and quantified by counting the tube branches.

### Western blotting

Cellular lysates were prepared from our prior study^43^. Proteins were resolved on SDS-polyacrylamide gel electrophoresis and then transferred to polyvinyldifluoride membranes. The blot membranes were blocked with 4% non-fat milk for 1 hr at room temperature, followed by incubation with primary antibodies at 4 °C for overnight. After washing three times, the blots were incubated with anti-rabbit or anti-mouse HRP-conjugated secondary antibodies for 1 hr at room temperature. Finally, the blots were visualized by enhanced chemiluminescence, using a Fujifilm LAS-3000 chemiluminescence detection system (Fujifilm; Tokyo, Japan)

### Quantitative real-time polymerase chain reaction (qPCR)

Total RNA was extracted from JJ012 cells by using TRIzol reagent. The messenger RNA was reversely transcribed to complementary DNA by using MMLV RT kit, and qPCR was then performed by using Taqman assay kit. The qPCR analysis of miR-27b expression was performed on StepOnePlus sequence detection system by using the TaqMan MicroRNA Reverse Transcription Kit and was normalized to U6 expression.

### Plasmid construction and luciferase reporter assay

Wild-type VEGF-C-3′-UTR was constructed into *pmirGLO* reporter vector between the *Nhe*I and *Xho*I cutting sites. The mutation of VEGF-C-3′-UTR was performed by Quickchange site directed kit (Stratagene; La Jolla, CA, USA) according to the manufacturer’s instructions.

To analysis the 3′-UTR luciferase activity, the JJ012 cells were transfected with wt-VEGFC-3′UTR or mt-VEGFC-3′UTR luciferase plasmids. Cells were lysed after 24 hr transfection, cell lysates were harvested and detected using luciferase assay system (Promega; Madison, WI, USA)

### Immunohistochemistry (IHC) staining

The human tissue sections were incubated with anti-VEGF-C (1:100) primary antibody at 4 °C overnight and then incubated with secondary antibody (1:100) for 1 hr at room temperature. Finally, the sections were stained with diaminobenzidine.

### Statistics

All quantified results are presented as the means ± SEM of at least three experiments. Statistical comparison of two groups was used the Student’s *t*-test. Statistical comparisons of more than two groups were used one-way ANOVA with Bonferroni’s post-hoc test. In all cases, *p* < 0.05 was defined statistically significant.

## Additional Information

**How to cite this article**: Yang, W.-H. *et al*. Leptin promotes VEGF-C production and induces lymphangiogenesis by suppressing miR-27b in human chondrosarcoma cells. *Sci. Rep.* **6**, 28647; doi: 10.1038/srep28647 (2016).

## Supplementary Material

Supplementary Information
