# Supplementary material for: Leptin promotes VEGF-C production and induces lymphangiogenesis by suppressing miR-27b in human chondrosarcoma cells
Source: Sci Rep. 2016 Jun 27;6:28647. doi: 10.1038/srep28647 (PMC4921910; doi:10.1038/srep28647)
Supplement: Supplementary Information [file srep28647-s1.doc]

**Leptin promotes VEGF-C production and induces lymphangiogenesis by suppressing miR-27b in human chondrosarcoma cells**

Wei-Hung Yang^1,2,3,4,#^, An-Chen Chang^5,#^, Shih-Wei Wang^6^, Shoou-Jyi Wang^7^, Yung-Sen Chang^1^, Tzu-Ming Chang^8^, Shao-Keh Hsu^8^, Yi-Chin Fong^9,10^* and Chih-Hsin Tang^11,12,13^*

^1^Department of Orthopedic Surgery, Taichung Hospital, Ministry of Health and Welfare, Taichung, Taiwan

^2^School of Chinese Medicine, China Medical University, Taichung, Taiwan

^3^Department of Nursing, National Taichung University of Science and Technology, Taichung, Taiwan

^4^Graduate Institute of Biotechnology, National Chung Hsing University, Taichung, Taiwan

^5^Institute of Biomedical Sciences, National Chung Hsing University, Taichung, Taiwan

^6^Department of Medicine, Mackay Medical College, New Taipei City, Taiwan

^7^Department of Orthopedic Surgery, Chang-Hua Hospital, Ministry of Health and Welfare, Puhsin Township, Changhua County, Taiwan

^8^Department of Orthopedic Surgery, Tungs' Taichung Metroharbor Hospital, Taichung, Taiwan

^9^Department of Sports Medicine, College of Health Care, China Medical University, Taichung, Taiwan

^10^Department of Orthopedic Surgery, China Medical University Beigang Hospital, Yunlin County, Taiwan

^11^Graduate Institute of Basic Medical Science, China Medical University, Taichung, Taiwan

^12^Department of Pharmacology, School of Medicine, China Medical University, Taichung, Taiwan

^13^Department of Biotechnology, College of Health Science, Asia University, Taichung, Taiwan

^#^ Co-first author

***Address correspondence to:**

Chih-Hsin Tang, PhD

Graduate Institute of Basic Medical Science, China Medical University, Taichung, Taiwan. No. 91, Hsueh-Shih Road, Taichung, Taiwan

Tel: 886-4-22052121-7726; Fax: 886-4-22333641; E-mail: [chtang@mail.cmu.edu.tw](mailto:chtang@mail.cmu.edu.tw)

Or

Yi-Chin Fong, MD, PhD

Department of Sports Medicine, College of Health Care, China Medical University

E-mail: [yichin.fong@gmail.com](mailto:yichin.fong@gmail.com)

**Supplementary data**


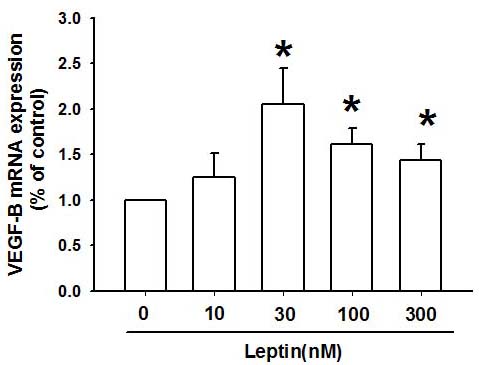


**Fig. S1. Leptin increases VEGF-B expression.** JJ012 cells were stimulated with leptin for 24 h, the VEGF-B mRNA level was measured by qPCR. The quantitative results were expressed as mean ± SEM. **P* < 0.05 as compared with control group.

.


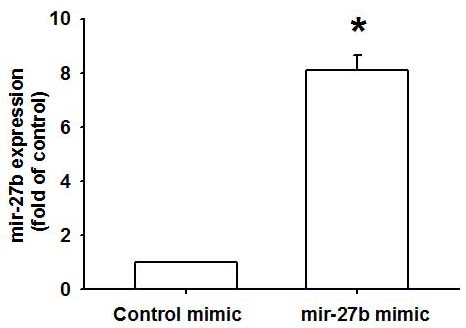


**Fig. S2. Mir-27b mimic enhances miR-27b expression.** JJ012 cells were transfected with indicated miRNA for 24 h, and miR-27b expression was measured by qPCR.
